# Protection against Cyclosporine-Induced Reprotoxicity by *Satureja khuzestanica* Essential Oil in Male Rats

**DOI:** 10.22074/ijfs.2015.4615

**Published:** 2015-12-23

**Authors:** Gholamreza Najafi, Farah Farokhi, Ali Shalizar Jalali, Zahra Akbarizadeh

**Affiliations:** 1 Histology and Embryology Research Laboratories, Department of Basic Sciences, Faculty of Veterinary Medicine, Urmia University, Urmia, Iran; 2 Department of Biology, Faculty of Science, Urmia University, Urmia, Iran

**Keywords:** Cyclosporine, Sperm, In Vitro Fertilization, *Satureja khuzestanica*

## Abstract

**Background:**

The effects of cyclosporine (Cs), a fungal cyclic polypeptide with potent immunosuppressive activity, on fertility have assumed greater significance with the increasing numbers of transplantations being performed all over the world. Current study was undertaken to investigate the potential of *Satureja khuzestanica* Essential Oil (SEO) as an antioxidant to mitigate Cs-induced reprotoxicity.

**Materials and Methods:**

In this experimental study (April-July 2012), thirty-two adult male Wistar rats were randomly divided into 4 groups of 8 animals each. Two groups of rats were administered Cs [40 mg/kg/day, per oral (p.o.)] for 45 days. One of these groups received SEO (225 mg/kg/day, p.o.) four hours after Cs administration. A vehicle-treated control group and a SEO control group were also included. Epididymal sperm characteristics, *in vitro* fertilizing capacity as well as embryo development were evaluated. For statistical analysis, one-way ANOVA and Tukey’s post-hoc test were used, and the value of P<0.05 was considered as the criterion for statistical significance.

**Results:**

Sperm count and viability along with fertilization and blastocyst development rates were significantly decreased by Cs treatment. Moreover, Cs-treated group showed significant increases in DNA damage, protamine deficiency of the sperm cells and proportion of spermatozoa with cytoplasmic droplet. Notably, aforementioned parameters were improved to near normal level by SEO co-administration.

**Conclusion:**

These results suggest that SEO has a protective action against Cs-induced reprotoxicity in a rat model.

## Introduction

Cyclosporine (Cs), a cyclic polypeptide with potent immunosuppressive activity, is a metabolite isolated from Tolypocladium inflatum and Cylindrocarpon lucidum (1). Cs has been widely used in transplant medicine and it has markedly improved graft survival rates in organ transplantation (2). Additionally, this drug is utilized in the treatment of various autoimmune disorders such as idiopathic nephritic syndrome (3), uveitis (4), psoriasis (5), rheumatoid arthritis (6) and inflammatory bowel disease (7). The primary immunosuppressive action of Cs is attributed to its inhibiting feature on the interleukin-2-interleukin-2-receptor autocrine pathway (8). Although Cs has enabled the success of clinical transplantation, its therapeutic application is limited by a number of adverse effects including renal, hepatic, cardiac, alimentary, neural and reproductive toxicity (9-14). Although the precise biochemical mechanism of Cs-induced toxicity in many organs is still a matter of debate, it has been shown that generation of reactive oxygen species (ROS) and membrane lipid peroxidation are causative factors involved in Cs-induced toxicities (15,17). Furthermore, it has been demonstrated that Cs induces reproductive toxicity through direct alterations in hypothalamic–pituitary-gonadal axis (18,20) and reduction in Sertoli cell phagocytic function (1). 

*Satureja khuzestanica*, belonging to the Lamiaceae (mint family), is an endemic plant of Iran that is known for its therapeutic application in folk medicine resulted from the essential oil existing in that (21). Different preparations of *Satureja khuzestanica* have been shown to have anti-inflammatory, antinociceptive, antimicrobial and antioxidant properties (22,24). Moreover, independent studies have revealed that *Satureja khuzestanica* Essential Oil (SEO) possess reproprotective and reproduction stimulatory properties (24,25). 

In view of this, since the antioxidative properties of SEO have been established, the present study was intended to evaluate the possible protective effect of SEO in experimental reprotoxicity induced in the rat model by Cs. 

## Materials and Methods

### Isolation of the essential oil

SEO was prepared from cultivated *Satureja khuzestanica* in Khoramabad in Lorestan province, western Iran. A dried voucher specimen was deposited at the Herbarium of the Botany Department, Faculty of Science, Urmia University, Urmia, Iran. The aerial parts of the plant were collected during the flowering stage, air-dried at ambient temperature in the shade and hydro-distilled using a Clevenger apparatus (Bakhshilab Co., Iran) for 4 hours, giving yellow oil in 0.9% yield. The oil was dried over anhydrous sodium sulfate and stored at 4˚C. The density of the essence was 0.94 (26). 

### Animals

For this experimental study (April-July 2012), thirty-two adult male albino rats of Wistar strain (4 months of age, 180-220 g body weight) were obtained from the Experimental Animal Production Center of the Science Faculty of Urmia University. All the animals were housed in special cages and had free access to tap water and to a pelleted commercial laboratory animal chow. Animal room temperature and relative humidity controls were set at 22 ± 2˚C and 50 ± 10%, respectively. Lighting was controlled to give a 12-hour light and dark cycle. All ethical themes of studies on animals were considered carefully and the experimental protocol was approved by the Ethics Committee for Research on Laboratory Animals at Urmia University. 

### Experimental design

After an adaptation period of 7 days, the animals were randomly divided into following four groups of 8 rats each as described below according to the treatment they received: control group, SEO group, Cs group and Cs+SEO group. The two experimental groups (Cs and Cs+SEO) were gavaged Cyclosporine (Sandimmune^®^, Novartis Pharmaceutical Corp., Switzerland) at a dose of 40 mg/kg/day dissolved in 0.5 mL olive oil. The controls were given an equivalent amount of olive oil. The SEO group was gavaged SEO at a dose of 225 mg/kg/day dissolved in 0.5 mL olive oil. The Cs+SEO group also received the same dose of SEO four hours after Cs administration. The treatment period was 45 days. The protocol for this study, including doses and duration of treatment for Cs and SEO, were all designed according to previous studies (27,29). 

### Sampling

Animals were euthanized by cervical dislocation following anesthesia with ketamine (Alfasan, Netherlands, 75 mg/kg, IP) 24 hours after the last treatment. The abdominal cavity was opened up through a ventral midline incision and epididymides were carefully separated from the testicles under a 20-time magnification provided by a stereo zoom microscope (Olympus, Japan). Testes and epididymides were then cleared of surrounding fat and connective tissues and weighed on a Mattler Basbal scale (Delta Range, Japan). 

### Epididymal sperm characteristic analysis

#### Epididymal sperm count

Epididymal sperm concentration was determined by a standard hemocytometer (HBG, Germany) as described previously (30). Briefly, one caudal epididymis was placed in 1 ml of rat 1-cell embryo culture medium (mR1ECM), chopped into 2-3 pieces and incubated for 10 minutes at 37˚C in an atmosphere of 5% CO_2_incubator to allow sperm to swim out of the epididymal tubules. After dilution of epididymal sperm to 1:20 in mR1ECM medium, approximately 10 μl of this diluted specimen was transferred to each of the counting chambers of the hemocytometer, which was allowed to stand for 5 minutes in a humid chamber to prevent drying. The sediment cells during this time were counted with a light microscope (Olympus, Japan) at ×400. The sperm count was expressed as number of sperm per milliliter. 

### Epididymal sperm viability

A 20 µl of sperm suspension was mixed with 20 µl of 0.05% eosin-yellowish (eosin-Y, Sigma-Aldrich, USA). Slides were assessed by a brightfield microscope with ×400 magnification following 2 minutes incubation at room temperature. Dead sperms appear pink and live sperms are not stained. In each sample 200 sperms were counted and viability percentages were recorded (31). 

### Epididymal sperm motility

In order to assess the sperm motility, 10 μl of the sperm suspension was placed on a clean prewarmed microscope slide and covered with a cover slip. At least 10 microscopic fields were examined at ×400 magnification using a light microscope equipped with a heated stage. The percentage of motile sperm was evaluated microscopically within 2-4 minutes of their isolation from the epididymides and expressed as a percentage of motile sperm of the total sperm counted (32). 

### Epididymal sperm DNA denaturation

Acridine orange (AO) assay was used to measure the susceptibility of cauda epididymal sperm DNA to acid-induced denaturation in experimental groups. To perform this assay with fluorescent microscope, thick smears were fixed in Carnoy’s fixative (methanol: acetic acid 1: 3) for at least 2 hours. The slides were stained for 5 minutes and gently rinsed with deionized water. About 200 sperms were evaluated, among which sperm heads with intact chromatin had green fluorescence, while those with denatured chromatin had orangered staining (33). 

### Epididymal sperm nuclear maturity

The acidic aniline blue (AB) stain is used to discriminate between lysine-rich histones and arginine/cysteine-rich protamines. This technique specifically provides a positive reaction with lysine residues in nuclear histones and reveals differences in the basic nuclear protein composition of the sperm. Histone-rich nuclei of immature sperms are rich in lysine and will consequently take up the blue stain. Protamine-rich nuclei of mature sperms are rich in arginine and cysteine and contain relatively low levels of lysine bringing about negative reaction to AB. The air-dried fixed smears were stained for 7 minutes with 5% AB in in phosphate buffered saline (PBS, Sigma-Aldrich, USA). The pH was adjusted to 3.5 using acetic acid. Slides were gently rinsed in distilled water and air dried. About 200 sperms per slide were counted under a light microscope (Olympus, Japan) using a ×100 oil immersion objective to determine the percentage of sperms stained with AB (34). 

### Cytoplasmic droplet count

Spermatozoa that retained their cytoplasmic droplet were enumerated by the methodology as reported previously (35). Sperm smears were prepared on clean and grease free slides, left to airdry overnight, stained with 1% eosin-Y/5% nigrosin and examined using an optical microscopy (Olympus, Japan). About 200 sperm cells per animal were examined to determine the percentage of sperms with cytoplasmic droplet. 

### In vitro evaluation of fertility potential and embryonic development

#### Collection of oocytes 

Female rats were injected subcutaneously with 25 IU pregnant mare serum gonadotrophin (PMSG, Folligon, Netherlands). Fifty-four hours later the rats were received an intraperitoneal injection of 20 IU human chorionic gonadotropin (hCG, Folligon, Netherlands). The rats were euthanized 19 hours after hCG injection. The oviducts were removed and each ampullar portion was placed into a plastic dish containing mR1ECM medium. Under a stereo zoom microscope (Olympus, Japan), 29-gauge insulin needle was used to gently tear open the swollen ampulla, allowing the oocytes in cumulus masses to extrude spontaneously into the medium (36). 

#### Sperm processing for in vitro fertilization and insemination

Sperm suspension were prepared and processed for *in vitro* fertilization (IVF) as described earlier. Spermatozoa were obtained by swim-up and incubated at 37˚C in an atmosphere of 5% CO_2_ for 1 hour to ensure capacitation. A volume of 0.1 mL of sperm suspension was introduced into 0.9 mL fertilization drop of mR1ECM medium containing oocytes from three females. For each animal, a total of 20 oocytes were divided into 10 drops. After six hours of incubation at 37˚C under 5% CO_2_, the cumulus cell free fertilized oocytes were transferred to fresh drops of mR1ECM medium for culture of embryos. All medium droplets were covered with mineral oil (37). 

#### Evaluation of fertilization rate

Twenty-four hours after insemination, oocytes were monitored by an inverted microscope and formation of the pronuclei and polar bodies was recorded to evaluate fertilization rate (FR). 

#### Blastocyst development rate determination

Blastocyst development rate (BDR) was evaluated by determining the number of embryos that had reached the blastocyst development stage after 72 hours incubation. Both late blastocysts (blastoceles greater than half the volume of the embryo) and expanding blastocysts (blastoceles that are fully expanded, with a thin zona pellucida) were included in the BDR (38). 

#### Measurement of lipid peroxidation

Lipid peroxidation (LPO) level in tissue homogenate was measured with the aid of a spectrophotometer. Malondialdehyde (MDA), which formed as an end product of the peroxidation of lipids, served as an index of LPO. MDA, referred to as thiobarbituric acid reactive substance (TBARS), was measured with TBA at 532 nm in a spectrophotometer (Biochrom, UK), as described previously (39). Results were expressed as nmol/g wet tissue. 

#### Data analysis

All data were expressed as the mean ± standard error of mean (S.E.M.). Differences between groups were assessed by one-way ANOVA using SPSS (SPSS Inc, USA) version 18.0. Statistical significance between groups was determined by Tukey’s post hoc test and the value of P<0.05 was used as the criterion for statistical significance. 

## Results

### Reproductive organ weights

The effects of Cs and SEO on relative testicular and epididymal weights are shown in table 1. Cs treatment produced a significant decrease in relative weights of testes and epididymides, indicating reproductive system damage, compared with the control group. These parameters rushed toward near normal levels of control in Cs+SEO-treated group.

**Table 1:** Effect of Cs and SEO on relative weights of testes and epididymides and epididymal sperm parameters (n=8)


	Control	Cs	SEO	Cs+SEO

Testes (weight/BW %)	0.89 ± 0.005	0.82 ± 0.003^a^	0.89 ± 0.006^b^	0.86 ± 0.003^a, b^
Epididymides (weight/BW %)	0.52 ± 0.005	0.45 ± 0.008^a^	0.53 ± 0.003^b^	0.49 ± 0.003^a, b^
Sperm count (106/ml)	50.50 ± 1.44	19.50 ± 0.86^a^	49.33 ± 1.45^b^	46.00 ± 1.15^b^
Sperm viability (%)	87.00 ± 1.73	68.00 ± 1.73^a^	86.33 ± 1.20^b^	79.50 ± 0.86^a, b^
Sperm motility (%)	79.33 ± 0.88	56.66 ± 0.88^a^	80.00 ± 1.73^b^	64.50 ± 0.86^a, b^
AO-positive sperms (%)	6.50 ± 0.86	28.00 ± 1.73^a^	5.83 ± 0.60^b^	10.50 ± 1.44^b^
AB-positive sperms (%)	8.00 ± 0.57	19.50 ± 0.86^a^	9.16 ± 0.44^b^	11.00 ± 2.30^b^


BW; Body weight, Cs; Cyclosporine, SEO; Satureja khuzestanica essential oil, AO; Acridine orange, AB; Aniline blue, ^a^; Statistically significant as compared with the control group at P<0.05 and ^b^; Statistically significant as compared with the Cs group at P<0.05. The values are expressed as mean ± SEM.

### Epididymal sperm parameters

**Fig.1:**
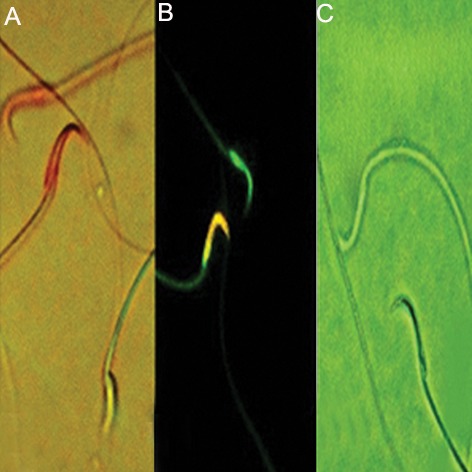
Photomicrographs of epididymal sperms in cyclosporinetreated rats. Epididymal sperms stained with eosin-Y: dead sperms appear pink and live sperm is not stained (×2000), B. Acridine orange stain to native DNA fluoresces green; whereas denatured DNA fluoresces orange-red (×2000) and C. Epididymal sperms stained with aniline blue: immature sperm head is blue; mature is unstained (×2000).

As shown in table 1, daily administration of Cs significantly reduced the mean epididymal sperm counts compared with their controls. There was also a significant decrease in the mean percentage of live and motile sperms of Cstreated rats compared with their control counterparts (Fig .1A). However, the simultaneous administration of SEO to Cs-treated animals significantly impeded a decrease in the epididymal sperm count and the percentage of live and motile sperms. 

Oral administration [per oral (p.o.)] of Cs brought about significant increase in the percentage of spermatozoa with DNA damage and chromatin abnormalities when compared to control rats (Fig .1B,C). On the other hand, co-administration of SEO caused significant decreases in sperm DNA damage and chromatin abnormalities respect to the Cs-treated group (Table 1). 

**Fig.2:**
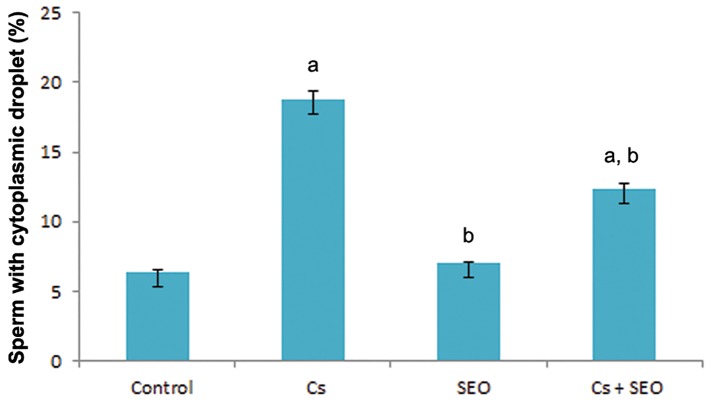
Effect of Cs and SEO on the percentage of spermatozoa that retain a cytoplasmic droplet (n=8). Cs; Cyclosporine, SEO; Satureja khuzestanica essential oil, a; Statistically significant as compared with the control group at P<0.05 and b; Statistically significant as compared with the Cs group at P<0.05.

The cytoplasmic droplet count for all groups is presented in figure 2. The percentage of spermatozoa that retained their cytoplasmic droplet was markedly higher in Cs-treated animals than those of the control animals, whereas co-treatment by SEO provided marked normalization in the percentage of spermatozoa having a cytoplasmic droplet when compared with the Cs group. 

### Fertilization rate

**Fig.3:**
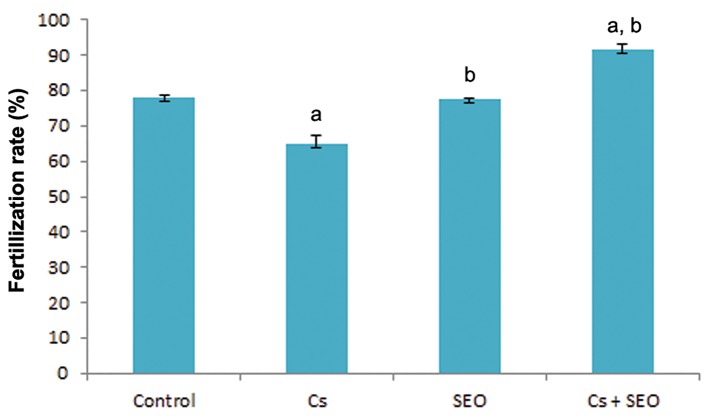
Effect of Cs and SEO on FR (n=8). Cs; Cyclosporine, SEO; Satureja khuzestanica essential oil, a; Statistically significant as compared with the control group at P<0.05 and b; Statistically significant as compared with the Cs group at P<0.05.

**Fig.4:**
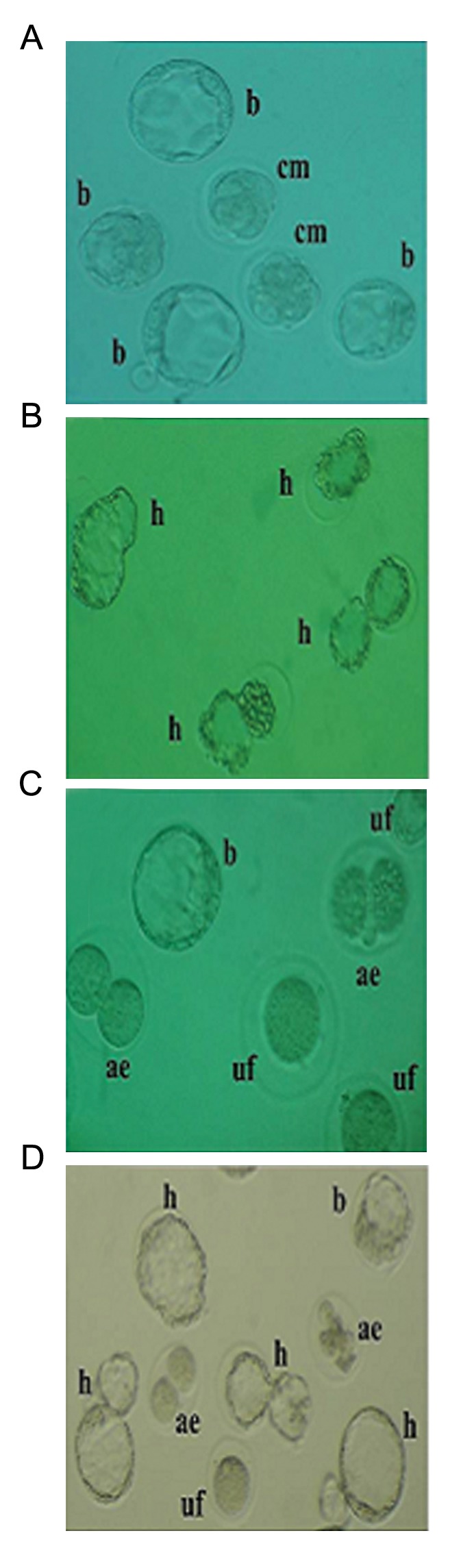
A. Representative photomicrographs of *in vitro* embryo development at 72 and 120 hours of culture from control, B. Satureja khuzestanica essential oil (SEO), C. cyclosporine (Cs) and D. SEO+CS treated rats. Differentiation to compact morula (cm), blastocyst (b) and hatched blastocyst (h) can be observed in control and SEO-treated groups. Cs administration alone caused significant increases in the rates of unfertilized oocytes (uf) and arrested embryos (ae). SEO co-treated animals exhibited nearly normal *in vitro* embryo development.

The effect of Cs and SEO on FR is depicted in figures 3 and 4. FR in animals exposed to Cs was significantly lower than control group, while treatment with SEO in combination with Cs increased the FR in rats compared with rats treated with Cs alone. Mean of fertile oocytes in control, Cs, SEO and Cs+SEO groups were 77.94% (152/195), 65.19% (148/227), 77.54% (145/187) and 91.80% (224/244), respectively. 

### Blastocyst development rate

**Fig.5:**
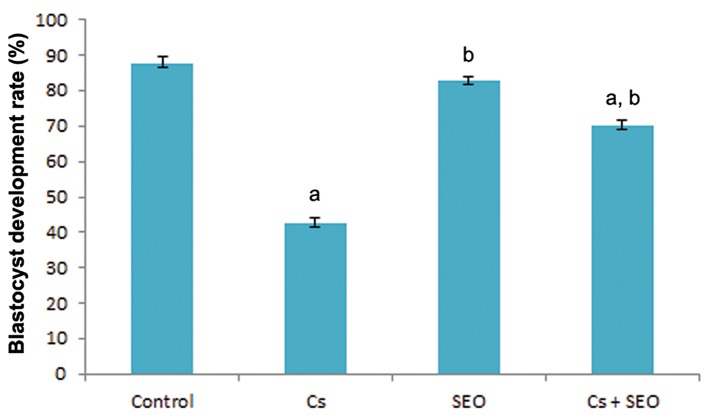
Effect of Cs and SEO on rat embryo development, expressed as the BDR (n=8). Cs; Cyclosporine, SEO; Satureja khuzestanica essential oil, BDR; Blastocyst development rate, a; Statistically significant as compared with the control group at P<0.05 and b; Statistically significant as compared with the Cs group at P<0.05.

The effects of different treatments on BDR are summarized in figures 4 and 5. Treatment with Cs alone resulted in a significant decrease in BDR, indicating embryotoxicity, as compared to the control. Administration of SEO along with Cs caused a significant improvement in this parameter compared to the Cs alone group. 

### Testicular tissue lipid peroxidation level

**Fig.6:**
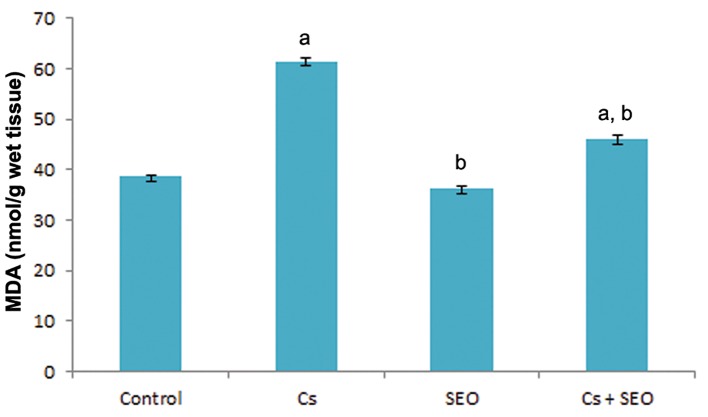
Effect of Cs and SEO on rat testicular tissue LPO (n=8). Cs; Cyclosporine, SEO; Satureja khuzestanica essential oil, MDA; Malondialdehyde, LPO; Lipid peroxidation, a; Statistically significant as compared with the control group at P<0.05 and b; Statistically significant as compared with the Cs group at P<0.05.

Testicular tissue LPO level, demonstrated as MDA, of all groups is given in figure 6. The MDA levels in the testicular tissue were found to be significantly higher in rats treated with Cs alone than those in the control group. The increase in MDA by Cs was significantly attenuated by SEO. 

## Discussion

Cs as an immunosuppressive of choice in transplant surgery has improved quality of life and survival of transplant patients (40). However, its clinical utility is accompanied by numerous unwanted side-effects such as reprotoxicity (18,20). Recently, there have been increasing interests in complementary medicine to alleviate Cs-induced toxicities in different organs (41,42). 

Several reports have demonstrated that enhancement of lipid peroxidation and oxidative stress (OS) following disruption in the oxidant–antioxidant status is the most possible biochemical mechanism of Cs-induced adverse effects in reproductive system (13,14,43). 

In the present study, reduction in weight of the testis and epididymis were indicative of Cs toxicity. Since the weight of the testes largely depends on the mass of the differentiated spermatogenic cells (44), the marked reduction in organ weight by Cs can be explained by diminished number of germ cells and a significant lower rate of spermatogenesis as confirmed by our findings. 

Mammalian spermatozoon as a highly differentiated haploid cell plays a crucial role in fertilization and its impairment is considered a major contributory factor to male infertility (45). Highly specific lipidic composition of mammalian sperm plasma membrane is responsible for its flexibility and the functional abilities of spermatozoa (46). However, substantial evidence indicates that mammalian spermatozoa are markedly vulnerable to the damages induced by enhanced, pathological ROS generation due to this unusual structure of sperm membrane (47). High levels of ROS production induce lipid peroxidation, a cascade of autocatalytic chemical reactions, in sperm cell membranes which can give a rise to cell dysfunction and death (48). 

In this study, Cs-treated rats showed significant decreases in epididymal sperm count and percentage of live sperm as compared to control animals. These findings are in agreement with previous reports at which was indicated that Cs causes spermatotoxicity through peroxidation of spermatozoa plasma membranes polyunsaturated fatty acids or alterations of the intracellular redox state (14,43). 

It has been established that protamines are involved in sperm chromatin stability as well as protection of spermatozoa against a variety of endogenous or exogenous stressors (49). A growing body of evidence indicates that OS-induced defects in spermatozoa maturation process can result in protamination disturbances leading to diminished sperm chromatin packaging which makes sperm cells more vulnerable to DNA damage (50,52). Moreover, extensive researches demonstrate that OS affects the integrity of the sperm genome by induction of high frequencies of DNA fragmentation (53,55). Consistent with above-noted findings, Cstreated animals in this study showed a significant decrease in the protamination of sperm chromatin as well as percentage of the sperms with doublestranded DNA in comparison to the control group. 

It is known that free radicals overproduction frequently involves in defective spermiogenesis leading to release of spermatozoa from the germinal epithelium carrying excess residual cytoplasm (56). It was also found that these immature, morphologically abnormal spermatozoa are the main source of further ROS generation in semen (57). Supporting these facts, our observations revealed that Cs treatment causes a marked elevation in the proportion of spermatozoa that retained their cytoplasmic droplet via mechanisms that may be mediated by OS. 

It is well documented that disturbances in the organization of the genomic material in sperm nuclei result in premature chromatin condensation leading to decreased fertilization rates and/or embryo cleavage (58,60). Additionally, earlier studies have linked high number of spermatozoa with DNA breaks and attached cytoplasmic droplets to fertilization and embryo development failures (61,62). In view of the fact that immature and structurally deficient spermatozoa are the major source of surplus free radicals production (63), FR and BDR reduction in Cs-administered rats may be attributed to the detrimental effects of ROS-producing damaged spermatozoa during *in vitro* insemination of oocytes. Our findings are confirmed by the study that reported *in vitro* incubation of oocytes with ROS-producing spermatozoa can result in impaired embryo development (64). 

Recently, there have been increasing interests in beneficial effects of antioxidants and naturally occurring substances against Cs-induced reproductive toxicity. Free radical scavengers such as ellagic acid have been shown to abate testicular damage and spermatotoxicity induced by Cs (43). Further, it has been reported that lycopene, an aliphatic hydrocarbon with highly efficient antioxidant capacity, provides protection against Cs-induced testicular toxicity through restoration of oxidant/antioxidant balance (14). Our previous study have also demonstrated that Crataegus monogyna fruit aqueous extract with prominent antioxidative property can effectively abate Cs-induced reproductive toxicities (65). 

In the present study, concomitant administration of SEO to Cs receiving rats markedly ameliorated the Cs-induced all the negative changes observed in the sperm parameters and embryo development, thereby highlighting its protective role in countering the oxidative injuries inflicted by free radicals. Consistent with our findings, prior studies have pointed out that SEO, as a strong and safe antioxidant, can protect from malathion and cyclophosphamide induced OS-mediated injurious effects (66,29). 

## Conclusion

Taken together, this study projects the protection afforded by SEO against OS-related reprotoxicity induced by Cs probably on the basis of oxidant-antioxidant system management. Further investigations are needed to explore therapeutic efficacy of SEO in clinical trials. 
